# Physical principles of membrane remodelling during cell mechanoadaptation

**DOI:** 10.1038/ncomms8292

**Published:** 2015-06-15

**Authors:** Anita Joanna Kosmalska, Laura Casares, Alberto Elosegui-Artola, Joseph Jose Thottacherry, Roberto Moreno-Vicente, Víctor González-Tarragó, Miguel Ángel del Pozo, Satyajit Mayor, Marino Arroyo, Daniel Navajas, Xavier Trepat, Nils C. Gauthier, Pere Roca-Cusachs

**Affiliations:** 1 Institute for Bioengineering of Catalonia (IBEC), Barcelona 08028, Spain; 2 Department of Physiological Sciences I, University of Barcelona, Barcelona 08036, Spain; 3 National Centre for Biological Sciences (TIFR), Bangalore 560065, India; 4 Centro Nacional de Investigaciones Cardiovasculares (CNIC), Madrid 28029, Spain; 5 LaCàN, Universitat Politècnica de Catalunya-BarcelonaTech, Barcelona 08034, Spain; 6 Ciber Enfermedades Respiratorias, Madrid 28029, Spain; 7 Institució Catalana de Recerca i Estudis Avançats (ICREA), Barcelona 08010, Spain; 8 Mechanobiology Institute, National University of Singapore, Singapore 117411, Singapore

## Abstract

Biological processes in any physiological environment involve changes in cell shape, which must be accommodated by their physical envelope—the bilayer membrane. However, the fundamental biophysical principles by which the cell membrane allows for and responds to shape changes remain unclear. Here we show that the 3D remodelling of the membrane in response to a broad diversity of physiological perturbations can be explained by a purely mechanical process. This process is passive, local, almost instantaneous, before any active remodelling and generates different types of membrane invaginations that can repeatedly store and release large fractions of the cell membrane. We further demonstrate that the shape of those invaginations is determined by the minimum elastic and adhesive energy required to store both membrane area and liquid volume at the cell–substrate interface. Once formed, cells reabsorb the invaginations through an active process with duration of the order of minutes.

## Abstract (web summary)

Variations in cell shape must be accommodated by the cell membrane, but how the membrane adjusts to changes in area and volume is not known. Here the authors show that the membrane responds in a nearly instantaneous, purely physical manner involving the flattening or generation of membrane invaginations.

## Graphical abstract

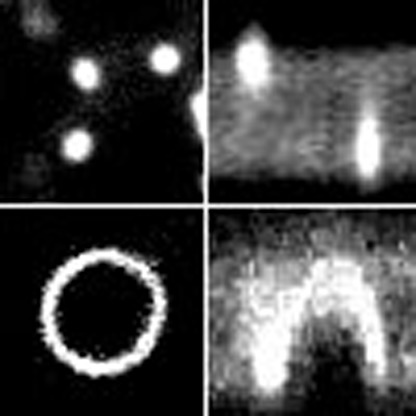

Physiological processes in development, wound healing, breathing or any other scenario generally involve cell shape variations, which are constrained by the physical envelope of cells—the plasma membrane. In any such process, the plasma membrane must adapt to often fast cell rearrangements, a requirement that is at odds with the very low membrane extensibility/compressibility given by its high stretching elastic modulus^1^,^2^. Other than simple extension and compression, the regulation of membrane area and shape therefore requires additional mechanisms, which could include active cell processes like endocytosis and exocytosis^3^,^4^,^5^ or the formation and flattening of membrane invaginations/evaginations, either at the micron scale as in membrane folds^6^,^7^, blebs^8^ or vacuole-like dilations (VLDs)^9^ or at the nanoscale as in caveolae^10^. However and despite extensive work on membrane mechanical interactions^11^,^12^,^13^,^14^,^15^, there is no clear physical understanding of the manner in which the cell membrane responds to changes in area and shape while remaining highly confined by adjacent cells or substrates.

Here we show that in response to changes in the area and volume of adherent cells, membrane remodelling occurs through a mechanical process that is passive, local, almost instantaneous and before any active response. This process generates invaginations with shapes that minimize the elastic and adhesive energy required to store both membrane area and liquid volume at the cell–substrate interface. Once formed, cells reabsorb the invaginations through an active process with duration of the order of minutes.

## Results

### Membrane response to changes in area and shape

**Figure 1:**
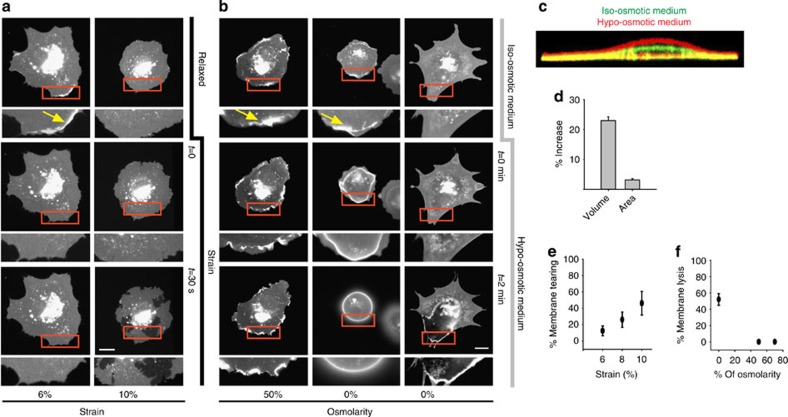
Membrane response to stretch and osmotic changes. (**a**) Cells transfected with pEYFP-mem before (top panel) and after (middle and bottom panels) applying different magnitudes of constant stretch. Yellow arrow indicates a membrane ruffle flattened by stretch. (**b**) Cells transfected with pEYFP-mem before and after reducing medium osmolarity to either 50 or 0% of original medium. Cells submitted to 0% osmolarity (de-ionized water) for 3 min sometimes rounded and flattened membrane ruffles (middle panel) and sometimes underwent membrane lysis (right panel). Yellow arrows indicate membrane ruffles, which either remain or flatten after applying 50 or 0% hypo-osmotic medium, respectively. (**c**) Confocal slice showing a cell before (green) and after (red) application of medium with 50% osmolarity for 3 min. (**d**) Corresponding quantification of the increase in cell volume and required membrane area (*n*= 5 cells). (**e**) % of cells showing membrane tearing after 3 min of constant stretch application (*n*=70 cells). (**f**) % of cells showing membrane lysis after 3 min of application of medium with different osmolarity (*n*=50 cells). Scale bars, 20 μm. Error bars are mean±s.e.m.

To understand how cell membranes respond to changes in area and shape, we labelled the membrane of mouse embryonic fibroblasts (MEFs) by transfection with a membrane fluorescent marker (pEYFP-mem) and seeded them on fibronectin-coated poly(dimethylsiloxane) (PDMS) membranes. We observed membrane dynamics after modifying two different cell shape parameters: cell volume (regulated through changes in medium osmolarity) and cell spreading area (regulated through a custom-built biaxial stretch device, Supplementary Fig. 1). After submitting cells to 6% linear strain (corresponding to a 12% increase in surface), we noted that additional required area was obtained by flattening membrane ruffles (Fig. 1a and Supplementary Movie 1). If stretch magnitude was increased, however, the membrane reservoir was depleted and the membrane teared within 3 min of constant stretch application (Fig. 1a,e and Supplementary Movie 1). In contrast, exposure to medium with a 50% reduction in osmolarity for 3 min increased cell volume by 20%, but only required an increase in plasma membrane area of 2% (Fig. 1c,d). By itself, this small increase in required area had a negligible effect on the membrane, as checked after stretching cells by 2% (Supplementary Fig. 2). Accordingly, 50% hypo-osmotic shock did not eliminate ruffles (Fig. 1b and Supplementary Movie 2), and exposure to 100% deionized water was required to eliminate membrane ruffles through cell swelling, or to lyse the membrane (Fig. 1b,f and Supplementary Movie 2).

**Figure 2:**
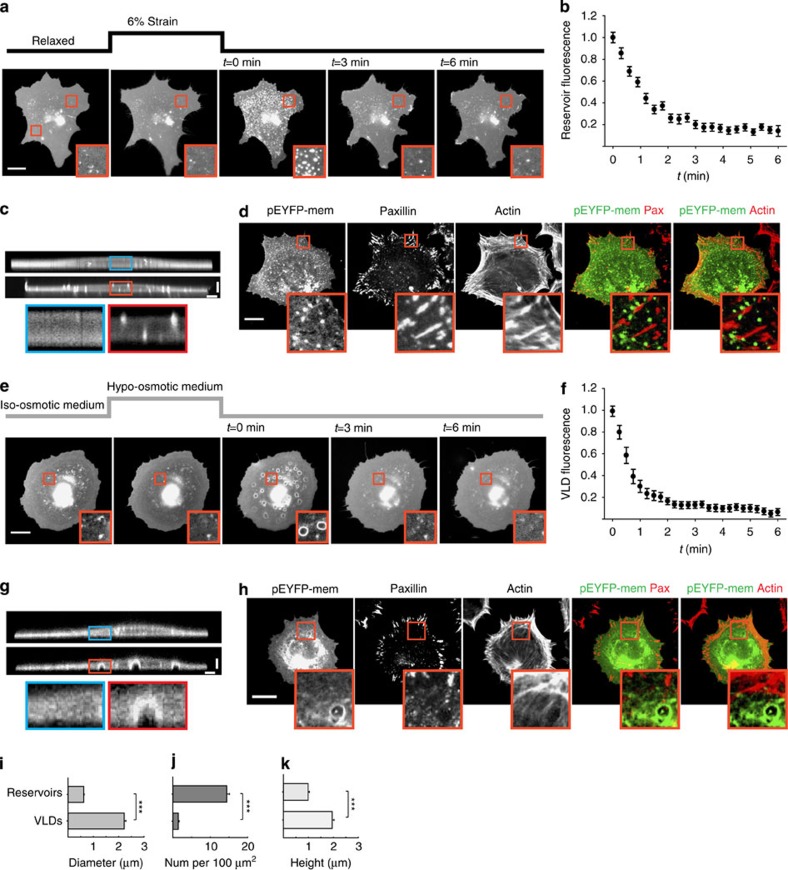
Cell membranes use different strategies to readapt to normal surface and volume. (**a**) pEYFP-mem-transfected cells before, during and after constant stretch application during 3 min. (**b**) Quantification of reservoir fluorescence after stretch release (1: initial fluorescence, 0: background). *n*=100 reservoirs from 10 cells. (**c**) Confocal vertical slice from a pEYFP-mem-transfected cell before (top) and after (bottom) application of 6% stretch for 3 min. (**d**) Staining images of cells fixed immediately after stretch release showing the membrane (pEYFP-mem transfection), paxillin and actin. Merged co-localization images are shown to the right. (**e**) pEYFP-mem-transfected cells before, during and after application of 50% hypo-osmotic medium during 3 min. (**f**) Quantification of VLD fluorescence after re-application of iso-osmotic medium (1: initial fluorescence, 0: background). *n*=100 VLDs from 10 cells. (**g**) Confocal images of a pEYFP-mem-transfected cell before (top) and after (bottom) application of 50% hypo-osmotic medium for 3 min. (**h**) Staining images of cells fixed immediately after re-application of iso-osmotic medium showing the membrane (pEYFP-mem transfection), paxillin and actin. Merged co-localization images are shown to the right. (**i**) Quantification of mean diameter of structures formed after stretch release (reservoirs) and re-application of iso-osmotic medium (VLDs). *n*=250/100 structures from 8/10 cells. (**j**) Quantification of mean density of structures formed after stretch release (reservoirs) and re-application of iso-osmotic medium (VLDs). *n*=30/50 regions from 5/8 cells. (**k**) Quantification of mean height of structures formed after stretch release (reservoirs) and re-application of iso-osmotic medium (VLDs). *n*=80/50 structures from 6/4 cells (***P<0.001, two-tailed Student's *t*-test). We note that reservoir heights are close to the axial resolution of our confocal microscope (0.9 μm) and thus represent upper estimates rather than accurate measurements. Scale bars are 5 μm in **c**,**g** and 20 μm in **d**,**h**. In all cases, zoomed insets (10 × 6 μm^2^ in **c**,**g** and 10 × 10 μm^2^ in **d**,**h**) show a magnification of the area marked in the main image. Error bars are mean±s.e.m.

After 3 min of stretch application, the release of stretch resulted in the accumulation of excess membrane in small membrane reservoirs of 0.5–1 μm in diameter (Fig. 2i–k), which extended from both the cell ventral and dorsal surfaces (Fig. 2a,c and Supplementary Movie 3). Through pEYFP-mem fluorescence quantification, we calculated that these reservoirs stored approximately 15% of the total projected cell–substrate membrane area (see methods), roughly matching the 12% change in area associated with 6% linear biaxial strain. Reservoirs were resorbed and eliminated by cells within ∼2 min (Fig. 2b), although their formation/resorption dynamics depended on temperature and stretch magnitude (see Supplementary Note 1 and Supplementary Fig. 3). Further, reservoirs appeared in open spaces devoid of actin fibres and focal adhesions, suggesting that membrane invaginations avoided cytoskeletal resistance (Fig. 2d).

Similarly, re-application of isotonic medium after 3 min of exposure to 50% hypo-osmotic medium resulted in the formation of membrane invaginations in the cell ventral surface, which were however larger (∼2 μm in diameter) and with a spherical cap shape (Fig. 2e,g and Supplementary Movie 3). The invaginations were quantified to store ∼2% of projected cell–substrate membrane area, thereby also matching the associated membrane requirement imposed by the osmotic shock (Fig. 1d). Those invaginations were also eliminated by cells within ∼3 min (Fig. 2f), and their formation/resorption dynamics depended on temperature and magnitude of hypo-osmotic shock (see Supplementary Note 1 and Supplementary Fig. 3). The membrane structures were concentrated at the central part of the cell, with a less dense actin meshwork and less focal adhesions, and their formation had to displace actin fibres and disrupt adhesions (Fig. 2h). This suggests that osmotically induced invaginations avoided sites of high cytoskeletal resistance like stretch-induced reservoirs, but due to their larger size also had to generate an opening through the cytoskeleton. The dynamic formation of those openings was confirmed by time-lapse images of cells co-transfected with both membrane and actin markers (Supplementary Fig. 4). The formation of such structures (termed VLDs) upon increases in medium osmolarity has long been described in neurons and other cell types^9^,^16^, and has been hypothesized to constitute a mechanism to accommodate excess membrane area upon osmotic-induced cell shrinking. However, 3-min incubation with 50% hypo-osmotic medium only imposed a 2% increase in membrane area (Fig. 1d), which by itself had negligible effects on the membrane (Supplementary Fig. 2). Thus, other factors beyond regulation of membrane area may drive VLD formation.

### VLD formation is driven by water confinement

**Figure 3:**
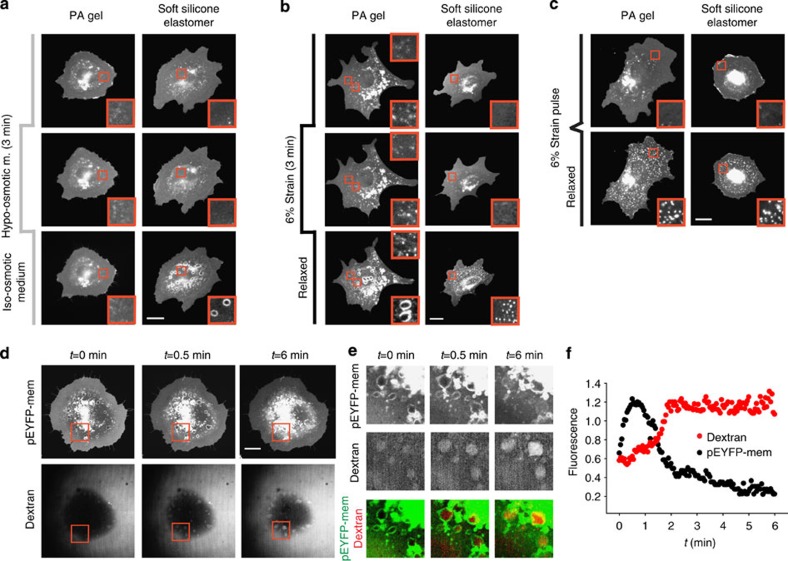
VLD formation is driven by the confinement of liquid flows at the cell–substrate interface. Response of pEYFP-mem-transfected cells seeded on either poly-acrylamide (PA) gels or soft silicone elastomers to: (**a**) the application of 50% hypo-osmotic medium for 3 min, (**b**) the application of 6% strain for 3 min and (**c**) a fast 6% strain pulse. Insets show zoomed views (10x10 μm^2^) of membrane structures. Scale bars, 20 μm. No significant differences were observed between any of the cases either in the diameter of reservoirs (*n*=150 reservoirs from 3 cells) or in their density (*n*=30 cell regions from 3 cells). (**d**) Time sequence of VLD formation and resorption in pEYFP-mem-transfected cells exposed to dextran-labelled iso-osmotic media after 3 min incubation with 50% unlabelled hypo-osmotic media. (**e**) Zoomed insets (20 × 20 μm^2^) corresponding to red square in **d** showing the evolution of membrane and dextran fluorescence, and merged images. (**f**) Corresponding quantification of pEYFP-mem and dextran relative fluorescence levels.

Alternatively to being regulated by membrane area, VLDs formed after increasing osmolarity could be caused by water flows exiting cells, which would be confined between cells and the substrate and thereby generate hydrostatic pressure. To test this, we seeded cells on polyacrylamide gels, through which water can flow. In those conditions, VLDs did not form upon the change from hypo- to iso-osmotic medium (Fig. 3a and Supplementary Movie 4). This effect was due to the water-permeable properties of polyacrylamide and not by its lower stiffness, as VLDs clearly formed in softer but hydrophobic silicone elastomers (Fig. 3a and Supplementary Movie 4). Interestingly, application of stretch for 3 min and subsequent release in cells seeded on polyacrylamide gels resulted in the formation not only of reservoirs as expected, but also of VLDs (Fig. 3b and Supplementary Movie 5). This was due to the poroelastic properties of polyacrylamide gels^17^, by which gels gradually swelled when stretched for 3 min, and then gradually released water and shrank upon stretch release (Supplementary Fig. 5). Confirming this, reservoirs but not VLDs formed on both soft silicone elastomers and polyacrylamide gels where swelling was prevented by submitting them to stretch only during a short pulse (Fig. 3c). Thus, water pressure formed either through confinement or poroelastic flows was equivalently successful at generating VLDs.

**Figure 4:**
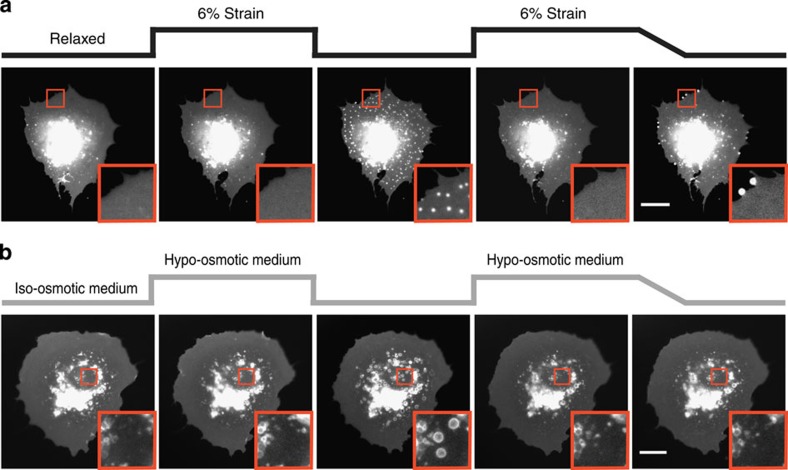
Effect of stimulus application rate on membrane structure formation. (**a**) Cell submitted to two successive steps of 6% stretch for 3 min, in which the first is released immediately and the second slowly (15 s). (**b**) Cell submitted to two successive applications of 50% hypo-osmotic media, in which iso-osmotic medium is restored first immediately and then slowly (1 min). Scale bars, 20 μm. Insets show zoomed views (10 × 10 μm^2^) of membrane structures.

We then evaluated further the degree of water confinement at the cell–substrate interface by submitting cells to a 3-min 50% hypo-osmotic shock, and then restoring iso-osmotic medium labelled with membrane-impermeable red fluorescent dextran. We did this at a low temperature of 26 °C, which slowed VLD formation and allowed us to distinguish the formation and resorption phases of VLD dynamics (see Supplementary Note 1). Whereas the medium surrounding cells immediately became fluorescent, the dextran-free water expelled by cells formed VLDs that had initially a very low fluorescence in the red channel (Fig. 3d,e). This shows that medium in VLDs was indeed confined, and did not immediately mix with external medium. However, as time progressed VLDs gradually increased their fluorescence even after VLDs started decreasing in size (Fig. 3e), demonstrating that water flow and mixing was impaired but not eliminated. Thus, the water pressure driving VLD formation was not caused by a complete seal, but by a transient and dynamic confinement generated by friction and flow restriction at the cell–substrate interface. Consistently, cells submitted to a gradual rather than sudden osmolarity increase had sufficient time to evacuate expelled water, preventing VLD formation (Fig. 4b). Interestingly, gradual rather than abrupt de-stretch also reduced reservoir formation, leading instead to membrane accumulations at the cell edge (Fig. 4a). This suggests that cells subjected to slow deformations can release membrane excess at locations where the membrane is not confined by a substrate, such as the cell edge. However, the increase in friction caused by fast de-stretch would prevent such long-scale rearrangements, forcing the membrane to release tension locally in reservoirs at the cell–substrate interface.

### Mechanism of membrane mechanical adaptation

**Figure 5:**
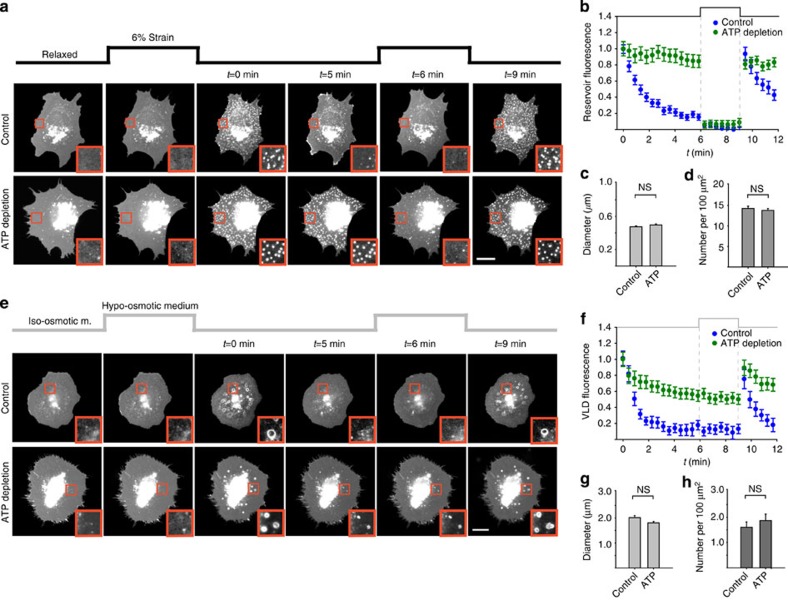
Membrane mechanical adaptation is a passive process followed by active recovery. (**a**) Examples of control and ATP-depleted pEYFP-mem-transfected cells before, during and after application of two 3-min constant stretch pulses. (**b**) Quantification of reservoir fluorescence after release of first stretch pulse and during application and release of the second pulse (*n*=50/70 reservoirs from 5/5 cells). The effect of ATP depletion was significant (*P*<0.001). (**c**) Quantification of reservoir size in control and ATP-depleted cells (*n*=150/200 reservoirs from 3/4 cells). No significant differences were observed, two-tailed Student's *t*-test. (**d**) Quantification of reservoir density in control and ATP-depleted cells (*n*=50/50 regions from 5/5 cells). No significant differences were observed. (**e**) Examples of control and ATP-depleted pEYFP-mem-transfected cells before, during and after application of 50% hypo-osmotic medium in two 3-min pulses. (**f**) Quantification of VLD fluorescence after the first re-application of iso-osmotic medium and during application and release of the second pulse (*n*=35/40 VLDs from 3/3 cells). The effect of ATP depletion was significant (*P*<0.001, two-tailed Student's *t*-test). (**g**) Quantification of VLD size in control and ATP-depleted cells (*n*=30/35 VLDs from 3/3 cells). No significant differences were observed. (**h**) Quantification of VLD density in control and ATP-depleted cells (*n*=20/25 regions from 3/3 cells). No significant differences were observed. Scale bars, 20 μm. Insets show zoomed views (10 × 10 μm^2^) of membrane structures.

In summary, reservoirs or VLDs were formed locally by mechanical stimuli imposing, respectively, a change in area (through stretch) or volume stored at the cell–substrate interface (through osmotic shocks or poroelastic flows, see Supplementary Note 2). We then evaluated different potential mechanisms to explain how those mechanical stimuli led to the formation of membrane structures. First, reservoirs and VLDs could be mediated by caveolae flattening, reported to occur in response to both stretch and hypo-osmotic shocks^10^. However, neither reservoirs nor VLDs co-localized with caveolin during our experiments, and both types of membrane structures still formed and resorbed in caveolin 1 knockout cells (Supplementary Fig. 6). Further, reservoirs and VLDs formed and resorbed equally in caveolin 1 knockout cells reconstituted either with caveolin 1-GFP or with an empty vector (Supplementary Fig. 6). Second, the membrane could adapt through any of the active ATP-dependent remodelling processes (such as exo- or endocytosis) regulating its area and shape^3^,^4^,^5^. However, whereas ATP depletion inhibited reservoir and VLD resorption, it did not affect their formation (Fig. 5a,e and Supplementary Movie 6). ATP depletion also inhibited the dynamic reservoir rearrangements that occurred during their resorption by cells (Supplementary Movie 6), showing that reservoir resorption but not formation is mediated by an ATP-dependent process. Similarly, actin cytoskeleton depolymerization with cytochalasin D or a reduction in temperature slowed the resorption of both reservoirs and VLDs, but did not prevent their formation (Supplementary Fig. 7). In addition, both reservoirs and VLDs were consistently observed across different cell types from diverse species (Supplementary Fig. 8). Thus, whereas both reservoirs and VLDs resorbed through an active actin- and temperature-dependent response, they formed by a general passive mechanical process. Further confirming the passive nature of reservoirs, their resorption in ATP-depleted cells could be induced by re-applying mechanical stretch (Fig. 5a,b and Supplementary Movie 6). Interestingly, whereas VLDs in ATP-depleted cells did not resorb, they gradually collapsed as water leaked from them (Fig. 5e,f and Supplementary Movie 7), leaving membrane accumulations similar to reservoirs. Those accumulations did not disappear upon re-application of hypo-osmotic medium, confirming (as observed in cells plated on polyacrylamide gels) that osmotic changes *per se* do not directly regulate membrane invaginations.

**Figure 6:**
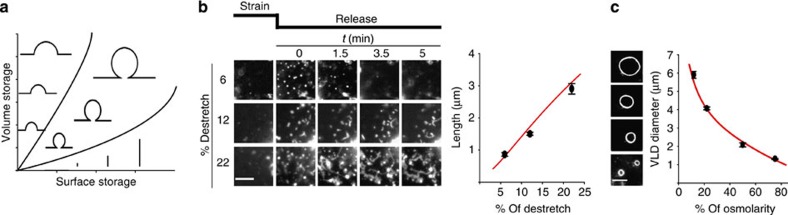
Membrane mechanical adaptation is explained by minimization of the strain and adhesion energies required to generate surface and volume containers. (**a**) Phase diagram showing the predicted structures that require minimal energy to deform the membrane and detach it from the substrate in order to accommodate membrane surface area (upon stretch release) and liquid volume at the cell–substrate interface (upon an increase in osmolarity)^18^. Surface storage is achieved optimally with increasingly long tubules, whereas volume storage leads to the formation of spherical caps (VLDs). When both volume and surface storage are required, spherical caps ‘bud' and become more invaginated. (**b**) Left: time-course sequences of cell membrane regions showing the formation of either reservoirs or increasingly long tubules after releasing different stretch magnitudes. Right: Mean reservoir/tubule length (black dots, experimental data, red line, theoretical prediction) as a function of de-stretch magnitude (for increasing stretch, *n*=80/50/50 structures from 6/3/3 cells). (**c**) Left: images showing the formation of increasingly large VLDs after restoring iso-osmotic medium in cells previously exposed to different magnitudes of hypo-osmotic shocks for 3 min. Right: mean VLD diameter (black dots, experimental data, red line, theoretical prediction) as a function of hypo-osmotic shock magnitude (for increasing osmotic shock, 60/100/100/50 structures from 5/5/10/3 cells). Scale bars, 5 μm. Error bars are mean±s.e.m.

Thus, the two types of membrane invaginations could apparently convert to each other, pointing at a unified framework of membrane mechanical adaptation. Given its passive nature, this framework could potentially mimic the behaviour of synthetic cell-free membrane systems. To explore this hypothesis, we adapted a theoretical approach (see Supplementary Note 3) previously shown to reproduce the behaviour of passive synthetic bilayer membranes adhered to a deformable substrate^18^. In this approach, membrane invaginations are understood as structures that store excess membrane area or interstitial volume with the least energy penalty. The energy sources considered are the elastic strain energy required to stretch and bend the membrane, and the adhesion energy required to detach the membrane from the substrate. This adhesion energy includes nonspecific interactions (as in the case of synthetic bilayers), but also specific bonds to the extracellular matrix mediated for instance by integrins. In the case of dorsal reservoirs substrate adhesion would not apply, but certain adhesion energy would still be required to detach the membrane from the underlying actin cortex. In this system, introducing liquid at the membrane–substrate interface results in the formation of VLD-like shallow spherical cap invaginations, which optimally store volume. In contrast, compressing the membrane results in the formation of tubular invaginations (with much higher surface/volume ratio), which optimally store excess membrane area. As surface/volume requirements increase, the model provides a phase diagram with increasingly large shallow caps to store volume, increasingly long tubules to store surface, and spherical invaginations with a small connecting neck to store both (Fig. 6a). If this framework applies to live cells, then the membrane reservoirs generated upon stretch release would in fact be short tubules, which should become longer for larger compressions. To test this, we seeded cells on pre-stretched membranes, and then further stretched the membrane. After 3 min, the total stretch (between 12 and 22%) was released, compressing the membrane. By using this two-step approach, we prevented the membrane tearing generally observed upon high stretch (Fig. 1). As expected, releasing stretch above 12% resulted in the formation of tubules, which became longer as stretch increased (Fig. 6b and Supplementary Movie 8). In some cases, tubules were observed to dynamically grow from reservoirs, showing that indeed reservoirs correspond to nascent membrane tubules (Supplementary Movie 8). Also as predicted by the model, VLD size, and therefore contained volume, increased with the magnitude of the hypo-osmotic shock (Fig. 6c).

**Figure 7:**
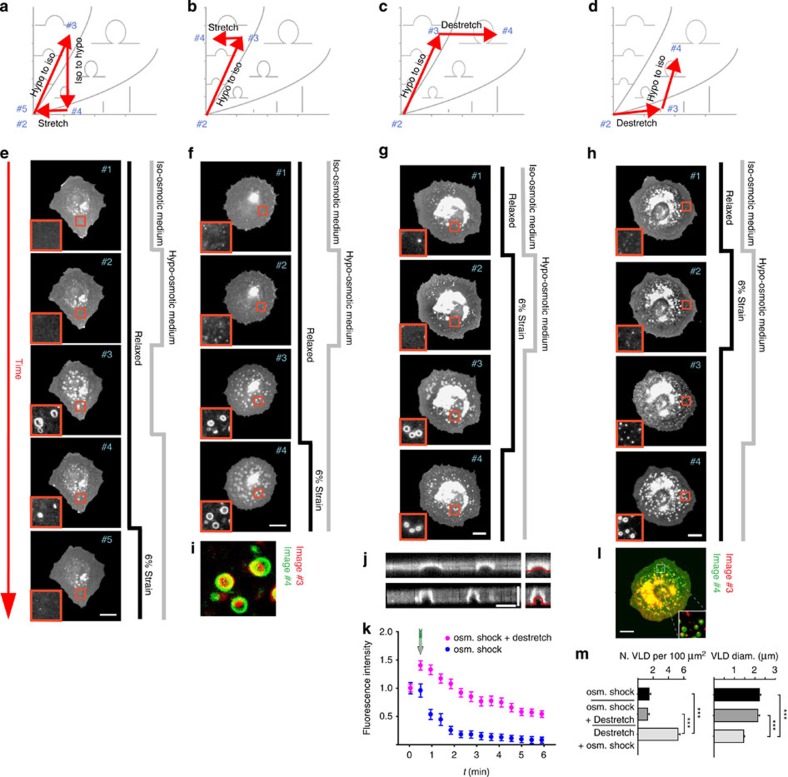
Membrane remodelling can be described through pathways along the surface/volume phase diagram. (**a**–**d**) Different pathways tested in the phase diagram by applying stretch and hypo-osmotic shocks (red arrows, numbers refer to the corresponding image in the panel below). (**e**–**h**) Corresponding response of pEYFP-mem-transfected cells after applying stretch and osmotic shocks as indicated to follow the pathways. Time flows from top to bottom. In all cases, the first application of stretch/hypo-osmotic shock (second row of cells) lasted 3 min. Subsequent steps were carried out as quickly as possible to evaluate membrane response before cells had time to actively eliminate structures. (**i**) In cells submitted to hypo-osmotic shock, co-localization of membrane structures formed after first restoring iso-osmotic medium (red) and then applying stretch (green). (**j**) Confocal vertical slices showing VLD shape before (top) and after (bottom) stretch release. Zoomed image to the right shows the superimposed shape prediction from the theoretical model in red. (**k**) Quantification of VLD fluorescence for cells under hypo-osmotic medium after either restoring iso-osmotic medium (blue symbols) or restoring iso-osmotic medium and then releasing stretch application (pink symbols, arrow indicates moment of stretch release). *N*=100/50 structures from 10/5 cells. (**l**) In cells submitted to both hypo-osmotic shock and stretch, co-localization of membrane structures formed after first releasing stretch (red) and then restoring iso-osmotic medium (green). (**m**) Quantification of VLD diameter (*n*=100/50/70 structures from 10/3/3 cells, ***P<0.001, analysis of variance (ANOVA)) and density (*n*=50/30/30 regions from 8/3/3 cells, ***P<0.001, ANOVA) in cells submitted only to osmotic shocks or also to de-stretch (stretch release) before or after restoring iso-osmolarity). Scale bars are 5 μm in **j** and 20 μm elsewhere. Insets show zoomed views (10 × 10 μm^2^) of membrane structures. Error bars are mean±s.e.m.

We then explored more complex membrane deformation pathways within the phase diagram (Fig. 7a–d). First, we generated VLDs in cells by decreasing and then restoring osmolarity, and then quickly re-applied hypo-osmotic medium. Cells quickly swelled and re-absorbed water in VLDs, leading to their immediate collapse (Fig. 7e) and confirming that hydrostatic pressure is key to their formation and maintenance (see Supplementary Note 2). However, mere removal of hydrostatic pressure was not sufficient to resorb membrane recruited upon VLD formation. As in ATP-depleted cells (Fig. 5e-f), bright membrane accumulations (‘collapsed' VLDs akin to reservoirs) remained at VLD sites (Fig. 7e). Subsequent application of stretch could then eliminate collapsed VLDs, closing the path in the phase diagram (Fig. 7a). In contrast, full non-collapsed VLDs were maintained by hydrostatic pressure and only increased in diameter upon stretch (Fig. 7f,i). Next, we submitted cells to both hypo-osmotic shock and stretch for 3 min and first restored iso-osmotic medium, leading to VLD formation as expected (Fig. 7g). Upon stretch release, excess membrane did not form reservoirs or tubules but rather accumulated at the site of VLDs, as indicated by a sharp increase in pEYFP-mem fluorescence (Fig. 7k). Confocal sections showed that VLDs became taller and more invaginated (Fig. 7j), in agreement with the structures predicted by the model to store both volume and membrane area (Fig. 7c). This latter finding highlights that pre-existing membrane invaginations (which are already bent and detached from the substrate) act as seeds for further membrane storage. To confirm this, we submitted cells to both stretch and hypo-osmotic medium and first released stretch, which resulted in reservoir formation (Fig. 7h). When we then restored iso-osmotic medium, VLDs indeed formed at the ‘seed' sites where reservoirs were previously located (Fig. 7l). As VLD formation was now dictated by the denser reservoir network, VLDs appeared in higher number and smaller size than those formed either in the absence of stretch or before stretch release (Fig. 7m).

Finally, we evaluated the effect of modifying one of the key parameters of the system, adhesion energy. To this end, we treated cells with a blocking antibody against α5β1 integrin, which we previously identified to provide adhesion strength to fibronectin-coated substrates in the same cell type^19^. Decreasing adhesion strength reduced the density of stretch-mediated reservoirs (Supplementary Fig. 9). Similar to the case of slow stretch release (Fig. 4), this suggests that reduced friction and interaction at the membrane–substrate interface allowed cells to release excess membrane in more distant but less confined membrane regions. In contrast, VLDs in cells with reduced adhesion slightly increased in density, and markedly increased in diameter (Supplementary Fig. 9). This is consistent with model predictions, as a reduction in adhesion would make it energetically favourable to detach a larger membrane area to generate each VLD. Inhibition of α5β1 also slowed VLD resorption, demonstrating that cells had an impaired ability to re-adhere detached membrane areas. In conclusion, the phase diagram provided by passive minimization of elastic and adhesive energies consistently predicted how the different perturbations generated membrane structures. The approximate dimensions of those structures, and their relative variations, were also correctly predicted after assuming parameter values consistent with experimental conditions (see Supplementary Note 3).

## Discussion

Despite extensive work, the mechanisms of membrane adaptation to physical constraints have remained elusive. It was recently shown that membrane area can be stored or released upon mechanical stimulation through the assembly/disassembly of caveolae^10^. However, the estimated 0.3% of membrane area contained in caveolae contrasts with membrane requirements of up to 10% for instance in spreading cells^7^, suggesting that additional buffers are required. Such buffers can be provided in time scales from seconds to minutes by active exocytic/endocytic processes^3^,^4^,^5^, which protect membrane integrity for instance in alveolar lung epithelial cells in response to both stretch^20^ and osmotic changes^21^. Here we show that, before the onset of any such active process, membranes adapt almost instantaneously through a passive process minimizing membrane elastic and adhesion energies, akin to what is observed in synthetic lipid membranes^18^,^22^. This process leads to the nucleation and growth of reservoirs/tubules to accommodate membrane area fractions that can be above 10%. The analogy between cell membranes and synthetic bilayers is surprising and has striking implications. First and addressing an unresolved issue^2^, our results suggest that if the perturbation is fast enough, membrane tension is released locally and not instantaneously transmitted across the cell (Fig. 4). The shape and size of membrane structures thus depends on both the magnitude and the dynamics of applied perturbations, which could lead for instance to the travelling membrane/cortex waves observed in cell-free surfaces^23^. Second and despite the complex molecular composition, cytoskeletal attachment and active behaviour of cell membranes^24^, we show that their mechanical adaptation can be successfully modelled by simply considering the two lipid layers. This is because the main mechanical parameter that constrains membrane deformation is the stretching modulus, which is determined by the membrane itself and not by the underlying actin cortex (see Supplementary Note 3). Finally, we note that reservoirs can store and release membrane area upon subsequent stretch cycles (Fig. 5), providing a regulatory mechanism potentially applicable to mechanical processes with time scales below the ∼2 min required for active membrane resorption (such as breathing, heart beating or muscle contraction).

Our results also demonstrate that VLDs, which were broadly understood as membrane area containers^4^,^9^,^25^, are driven instead by hydrostatic pressure from water stored at the cell–substrate interface. Their role in processes such as cell adaptation to shrinking should therefore be revisited, as cells in most physiological settings will be surrounded by permeable extracellular matrices, which would not constrain water flow from cells. However, we show that VLDs can be equivalently formed by hydrostatic pressure arising from other sources (such as poroelastic flows, Fig. 3b), suggesting a significant role in the cellular adaptation to any excess water pressure at the cell–matrix interface. The fact that increasing osmolarity leads to the immediate formation of VLDs rather than merely expelling water through the dorsal surface also confirms that the cytoplasm exhibits limited water mobility and slow pressure redistribution^26^. Both reservoirs/tubules and VLDs form at sites of low cytoskeletal density and resorb through an actin- and ATP-dependent process. This active cell response likely involves actin polymerization to push the lamellipodium and re-stretch and flatten the cell membrane, endocytic processes to detach invaginated reservoirs or tubules, or vesiculation, that is, the detachment of membrane vesicles from tubules that has been observed both in live cells^27^ and in passive bilayer systems^18^. However and regardless of the specific active mechanisms by which cells re-absorb membrane structures, the physical principles that drive their formation may themselves be harnessed by cells to respond to changes in cell shape arising in any instance of cell migration or deformation. Further, the biochemical activity of membrane curvature-sensitive molecules^2^,^28^,^29^ could also be affected by the local curvature induced by tubules or VLDs, potentially initiating mechanotransduction cascades.

## Methods

### Cell culture and reagents

MEFs were previously described^19^,^30^ and cultured in DMEM supplemented with 10% fetal bovine serum (FBS). One day before experiments, cells were transfected with the membrane-targeting plasmid pEYFP-mem (Clontech) or lifeact-ruby using the Neon transfection device according to the manufacturer's instructions (Invitrogen). pEYFP-mem contains the N-terminal 20 amino acids of neuromodulin, which is palmytoylated post-translationally and targets the EYFP fluorophore to membranes. Cells were incubated with cytochalasin D for 30 min to depolymerize the actin cytoskeleton (0.5 μM, Sigma) and with 10 mM deoxy-D-glucose plus 10 mM NaN_3_ (Sigma) to deplete ATP levels. Dextran experiments were carried out with 0.5 mg ml^−1^ of tetramethylrhodamine-labelled dextran (10,000 MW, Life technologies), and α5β1 integrins were blocked with 10 μg ml^−1^ α5β1 antibody (Merck Millipore, clone BMB5). The role of caveolin 1 was analysed by using Caveolin1 Knock out MEFs reconstituted with Cav1 or IRES-GFP as a control^31^. Cav1 was cloned in the lentiviral vector pRR SIN 18 CMV IRES EGFP. To generate these stable cell lines, the infected cells were selected by sorting for GFP marker expression by flow cytometry (FACS). Cav1 expression was checked by western blot analysis and reverse transcription–quantitative PCR. Cells expressing levels of Cav1-GFP comparable to endogenous Cav1 levels in wild-type MEFs were selected for experiments. Chinese Hamster Ovary cells were cultured in HF-12 medium supplemented with 10% FBS. Human keratinocytes (HaCaT) were cultured in DMEM supplemented with 10% FBS. Human squamous carcinoma cells (A431) were cultured in Earle's Balanced Salt Solution supplemented with 10% FBS.

### Preparation of stretchable membranes

The stretchable PDMS membranes (see schematic in Supplementary Fig. 1) were prepared by mixing the PDMS base and crosslinker at a 10:1 ratio, degassing for 1 h, spinning the mixture on a 13-cm sheet (500 r.p.m., 1 min) and curing at 65 °C overnight. Once cured, PDMS membranes were peeled off and placed tightly between the rings of the stretching device (Supplementary Fig. 1). Membranes were then coated with 10 μg ml^−1^ fibronectin (Sigma) overnight at 4 °C, or attached to either polyacrylamide or soft silicone elastomers. To attach polyacrylamide gels to membranes^32^, gels were prepared by using a mixture of 10% acrylamide and 0.3% bis-acrylamide and polymerizing between two coverslips treated with repel-silane (Young's modulus ∼30 kPa). Once polymerized, one coverslip was removed and the gel was pressed in contact with the PDMS membrane, which had previously been treated with 3-aminopropyl triethoxysilane 10% in ethanol for 1 h at 65 °C and with glutaraldehyde (1,5%) in PBS for 25 m at room temperature. After overnight incubation at 37 °C in a humid chamber for covalent binding, the other coverslip was removed and the gel was incubated with 10 μg ml^−1^ fibronectin overnight at 4 °C, resulting in membrane-attached gels ready for cell culture. Soft silicon elastomers (CY 52–276, Dow Corning, with Young's modulus ∼8 kPa (ref. 33)) were prepared by mixing CyA and CyB components at a 1:1 ratio and curing at 80 °C for 2 h (ref. 33). The substrates were then attached to membranes following the same procedure as for polyacrylamide gels. In some experiments not involving stretch, PDMS membranes were cured directly on glass coverslips instead of placing them in the stretch system.

### Stretch and osmolarity experiments

Once PDMS membranes were either directly coated with fibronectin or covalently attached to fibronectin-coated polyacrylamide/soft silicone gels, cells were seeded on the membrane and allowed to spread in the incubator for 0.5 h. Then, membranes were placed on the stretch system (Supplementary Fig. 1), consisting of a central loading post and an external ring. Vacuum was then applied through the space between the loading post and the external ring, thereby deforming and stretching the membrane. Cells spread on the membrane directly on top of the central loading post experienced an equibiaxial strain which depended on the vacuum pressure applied (Supplementary Fig. 1). The system was then mounted on the microscope stage. To modify osmolarity, cells were exposed to medium mixed with de-ionized water in which the concentrations of Ca^2+^ and Mg^2+^ had been corrected.

### Imaging

Cell images of fluorescently labelled cells were obtained using an upright microscope (Nikon eclipse Ni-U) with a water immersion objective ( × 60 magnification, NA=1.0) and an Orca Flash 4.0 camera (Hamamatsu). To obtain three-dimensional stacks, an inverted microscope (Nikon Eclipse Ti) with a spinning disk confocal unit (CSU-W1, Yokogawa), a Zyla sCMOS camera (Andor) and a × 60 objective was used. This objective was either of oil immersion (NA=1.42) or of water immersion (NA=1.0) for experiments involving stretch, as viscous oil droplets dragged the flexible PDMS membrane used for stretch and precluded proper focusing.

### Analysis of membrane structures

The evolution of membrane structures (reservoirs and VLDs) was analysed by measuring the time course of the pEYFP-mem fluorescence of each structure. To correct for photobleaching, this fluorescence was expressed as the fold-increase with respect to the background fluorescence of neighbouring cell regions without membrane structures, and normalized between 1 (initial fluorescence after stretch release or osmolarity increase) and 0 (background cell fluorescence). The diameter and density of structures was also measured, and the height was obtained from confocal slices. We note, however, that reservoir heights are close to the axial resolution of our confocal microscope (0.9 μm) and thus represent upper estimates rather than accurate measurements. The membrane fraction contained in membrane structures was estimated by comparing the average pEYFP-mem fluorescence of cell regions containing structures to the average fluorescence of structure-free zones within the same region. To ensure that we only considered the fluorescence of structures induced by stretch or osmotic shocks, the analysis was carried out in regions devoid of visible endomembrane structures before the application of stretch or osmotic shocks. In all time-lapse fluorescence images shown in figures, contrast was adjusted in each image to correct for the effect of photobleaching and leave cell background at a uniform level. Supplementary Videos show the full time-lapse videos without this adjustment, thereby showing the effect of photobleaching. The videos also show a decrease in fluorescence upon application/release of stretch, caused by added photobleaching during the re-centering and re-focusing of cells after their movement.

### Cell volume and surface estimations

Changes in cell volume and required membrane surface were calculated from spinning disk confocal slices obtained in cells before and after a 3-min 50% hypo-osmotic treatment. Cells were first re-sliced in the *XZ* plane (resulting in the images observed for instance in Fig. 2g), and membrane fluorescence images were binarized by thresholding. Cell volume was then calculated by adding the total number of pixels inside cells from all slices and multiplying by voxel size. To measure membrane surface, the cell perimeter in *XZ*-plane confocal slices was drawn manually. This avoided spurious increases in the estimated cell perimeter caused by the jagged cell edge resulting from binarization. We note that our area estimates do not correspond to the total membrane area, which may contain small folds not resolved by microscope images. Rather, our measurements estimate the increase in membrane area required to accommodate the global change in shape produced by cell swelling.

### Immunostaining

For fluorescence staining, cells were fixed with 4% paraformaldehyde, permeabilized with 0.1% Triton X-100 and labelled first with primary antibodies (2 h, room temperature), and then with Alexa-conjugated secondary antibodies (Invitrogen, 2 h, room temperature). Primary antibodies used were against paxillin (2 μg ml^−1^, clone 349 produced in mouse, ref. 610051 from BD Transduction Laboratories) and Caveolin 1 (4 μg ml^−1^, CAV1, polyclonal antibody produced in rabbit, ref. 610060 from BD Transduction Laboratories). Phalloidin-Tetramethylrhodamine B isothiocyanate (Sigma) was used instead of primary antibodies to label actin.

### Modelling

Theoretical modelling was carried out using a previously described approach^18^. Model assumptions, parameters and predictions are described in Supplementary Note 3.

### Stastistical analysis

Statistical comparisons were carried out with two-tailed Student's *t*-tests when two cases were compared and with analysis of variance tests when more cases were analysed. All data are shown as mean±s.e.m.

## Additional information

**How to cite this article:** Kosmalska, AJ. *et al.* Physical principles of membrane remodelling during cell mechanoadaptation. *Nat. Commun.* 6:7292 doi: 10.1038/ncomms8292 (2015).

## Supplementary Material

Supplementary Information Supplementary Figures 1-10, Supplementary Notes 1-3 and Supplementary References.

Supplementary Movie 1 Time sequence of YFP-mb transfected cells before and after stretching by 6% (top) or 10% (bottom). Scale bar indicates 20 μm. Zoomed areas to the right show an enlarged view of the zones marked with a white square.

Supplementary Movie 2 Time sequence of YFP-mb transfected cells before and after decreasing osmolarity to either 50% (top panel) or 0% (middle and bottom panels). Scale bar indicates 20 μm. Zoomed areas to the right show an enlarged view of the zones marked with a white square.

Supplementary Movie 3 Time sequence of YFP-mb transfected cells before, during, and after submitting them for three minutes to either 6% strain (top) or a 50% hypo-osmotic shock (bottom). Scale bar indicates 20 μm. Zoomed areas show an enlarged view of the zones marked with a white square.

Supplementary Movie 4 Time sequence of YFP-mb transfected cells before, during, and after submitting them to a 50% hypo-osmotic shock for three minutes. Cells were seeded either on a polyacrylamide gel (top) or a soft silicone elastomer (bottom). Scale bar indicates 20 μm. Zoomed areas show an enlarged view of the zones marked with a white square.

Supplementary Movie 5 Time sequence of YFP-mb transfected cells before, during, and after submitting them to a 6% strain for three minutes. Cells were seeded either on a polyacrylamide gel (top) or a soft silicone elastomer (bottom). Scale bar indicates 20 μm. Zoomed areas show an enlarged view of the zones marked with a white square.

Supplementary Movie 6 Time sequence of YFP-mb transfected cells submitted to two consecutive steps of three-minute 6% strain application and subsequent stretch release. Top cell, control, bottom cell, ATP depletion. Scale bar indicates 20 μm. Zoomed areas show an enlarged view of the zones marked with a white square.

Supplementary Movie 7 Time sequence of YFP-mb transfected cells submitted to two consecutive steps of three-minute application of 50% hypo-osmotic media and subsequent re-application of iso-osmotic media. Top cell, control, bottom cell, ATP depletion. Scale bar indicates 20 μm. Zoomed areas show an enlarged view of the zones marked with a white square.

Supplementary Movie 8 Time sequence of YFP-mb transfected cells during the application and subsequent release of 12% strain (top) and 21% strain (bottom). Scale bar indicates 20 μm. Zoomed areas show an enlarged view of the zones marked with a white square
