# Supplementary material for: Physical principles of membrane remodelling during cell mechanoadaptation
Source: Nat Commun. 2015 Jun 15;6:7292. doi: 10.1038/ncomms8292 (PMC4490354; doi:10.1038/ncomms8292)
Supplement: Supplementary Information — Supplementary Figures 1-10, Supplementary Notes 1-3 and Supplementary References. [file ncomms8292-s1.pdf]

Supplementary figures

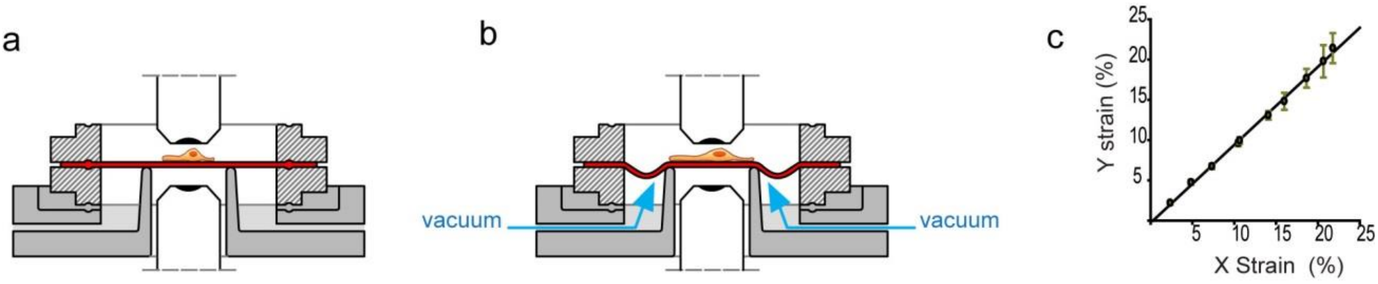

Supplementary Figure 1. Stretch system. a, Schematic of stretch system. A ring clamping a PDMS membrane (shown in red) is placed on a support containing a central loading circular post and an external ring, with an opening in between. Imaging objectives can then be placed either below (inverted microscope) or above (upright microscope). Cells are then cultured on the membrane after coating with fibronectin. **b**, Once vacuum is applied through the opening, it deforms and stretches the membrane. In some cases, cells were seeded on polyacrylamide and soft silicone gels previously attached to the membrane (see methods). **c**, Level of strain in the X and Y axis obtained after applying different vacuum suction pressures. Stretch was biaxial.

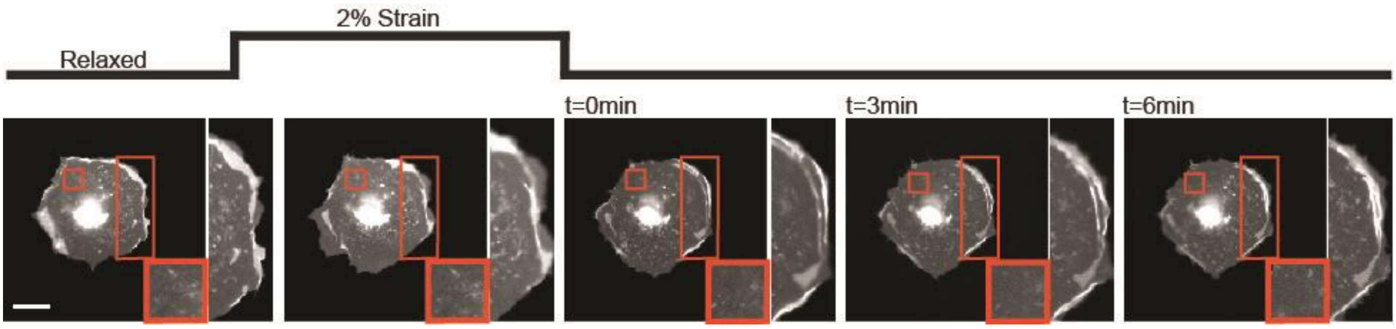

Supplementary Figure 2. Response of cells to 2% strain. Response of a peYFP-mem transfected cell to the application of 2% strain for three minutes. No visible effects were observed either upon stretch application or upon stretch release. Insets display magnifications of the areas in red, showing that membrane ruffles were not flattened upon stretch application, and reservoirs were not formed upon stretch release. Scale bar is 20 µm.

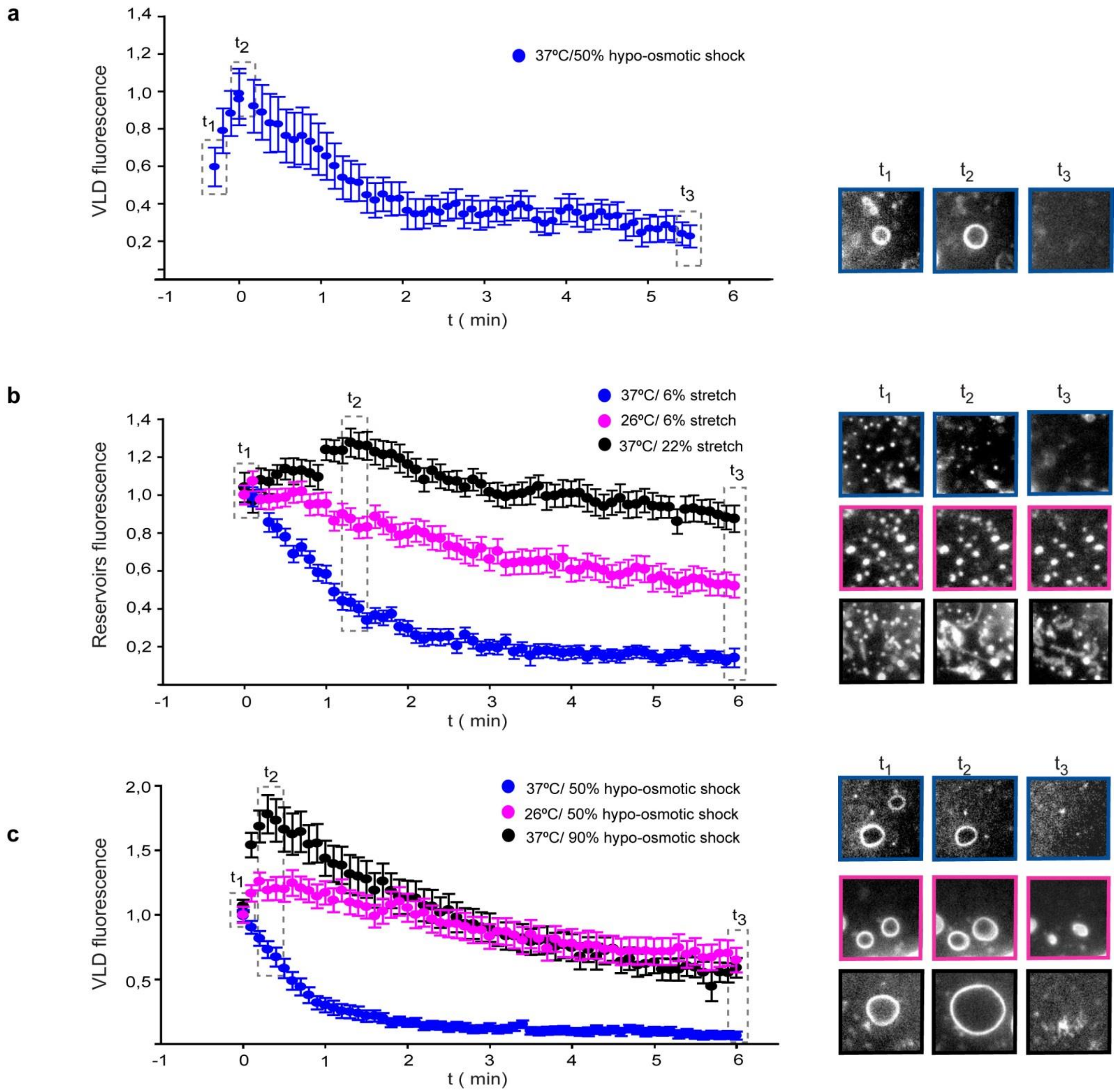

Supplementary Figure 3. Dynamic formation and resorption of membrane structures. a, Quantification of VLD fluorescence after re-application of iso-osmotic medium (1:maximum fluorescence, 0: background). n= 20 VLDs from 2 cells. Imaging was carried out using an inverted microscope (60x objective) which allowed to visualize the initial VLD formation period. **b**, Quantification of reservoir fluorescence after stretch release (1: initial fluorescence, 0: background). n= 100/80/30 reservoirs from 10/3/3 cells. Imaging was carried out using an upright microscope (60x objective). Both decreasing temperature (pink symbols) and increasing stretch magnitude (black symbols) slowed the process of reservoir formation. Images to the right show corresponding examples of membrane structures at the times indicated in the graph. **c**, Quantification of VLD fluorescence after re-application of iso-osmotic medium (1: initial fluorescence, 0: background). n= 100/50/60 VLDs from 10/5/4 cells. Imaging was carried out using an upright microscope (60x objective). Both decreasing temperature (pink symbols) and increasing the magnitude of the hypo-osmotic shock (black symbols) slowed the process of VLD formation. Images to the right show corresponding examples of membrane structures at the times indicated in the graph. All insets have a size of 10x10 µm.

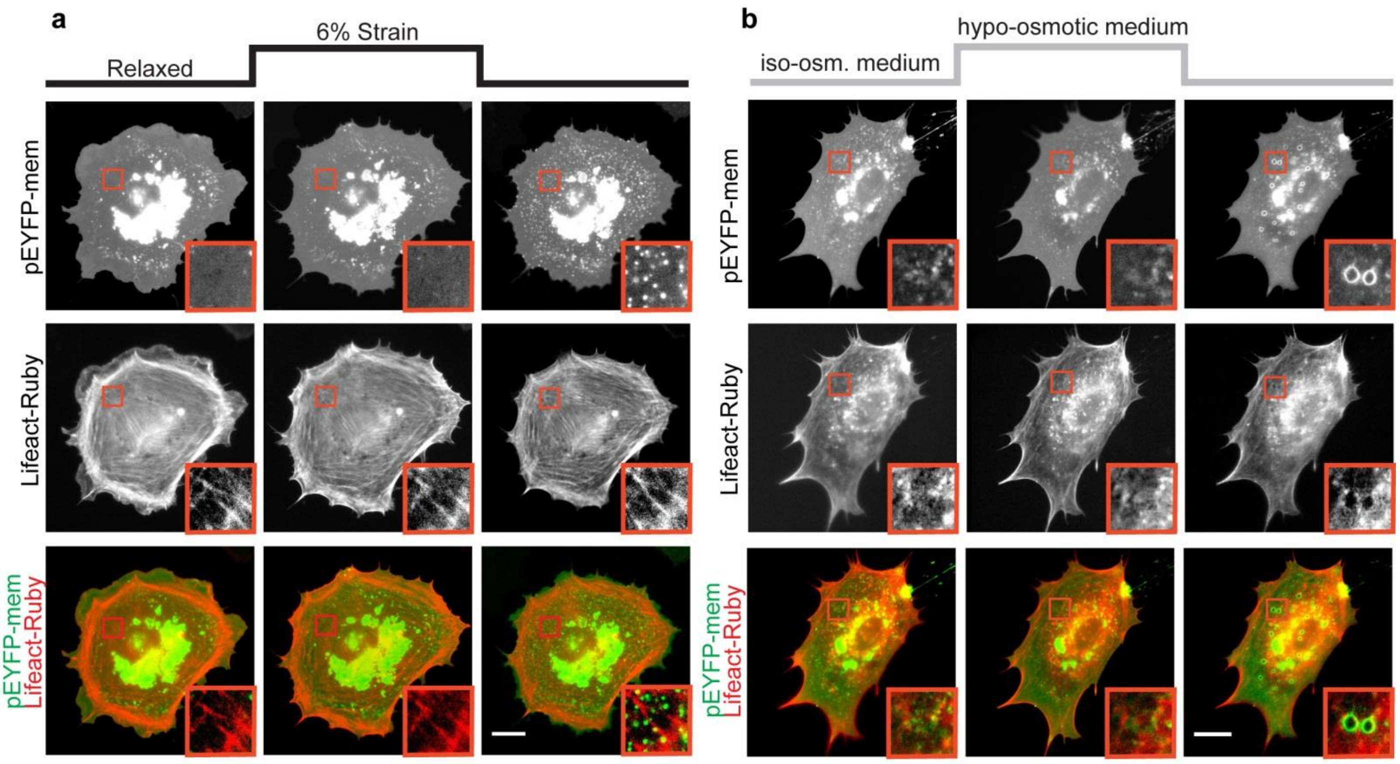

Supplementary Figure 4. Co-localization of actin and membrane structures before, during and after stretch and hypo-osmotic shocks. a, cells transfected with pEYFP-mem and Lifeact-Ruby before, during, and after application of 6% stretch for 3 minutes. **b**, equivalent images obtained before, during, and after application of a 50% hypo-osmotic shock for 3 minutes. Insets (10x10 µm) show zoomed views of membrane and actin structures**.** Scale bars are 20µm.

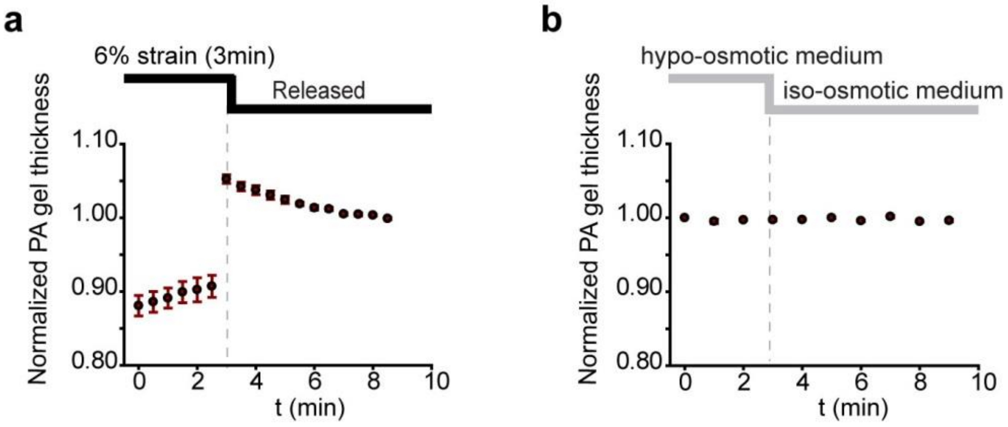

Supplementary Figure 5. Poroelasticity of polyacrylamide gels. a, graph showing the height of polyacrylamide gels during and after application of 6% stretch for three minutes. Values are shown normalized to the height before stretch application. As gels are stretched, they first decrease their height to maintain volume, but they progressively incorporate water and swell. Once stretch is released, the progressive reduction in thickness is indicative of water expulsion flow. **b**, As a control, gels submitted to a 50% decrease in media osmolarity did not modify their height. (n=5 gels)

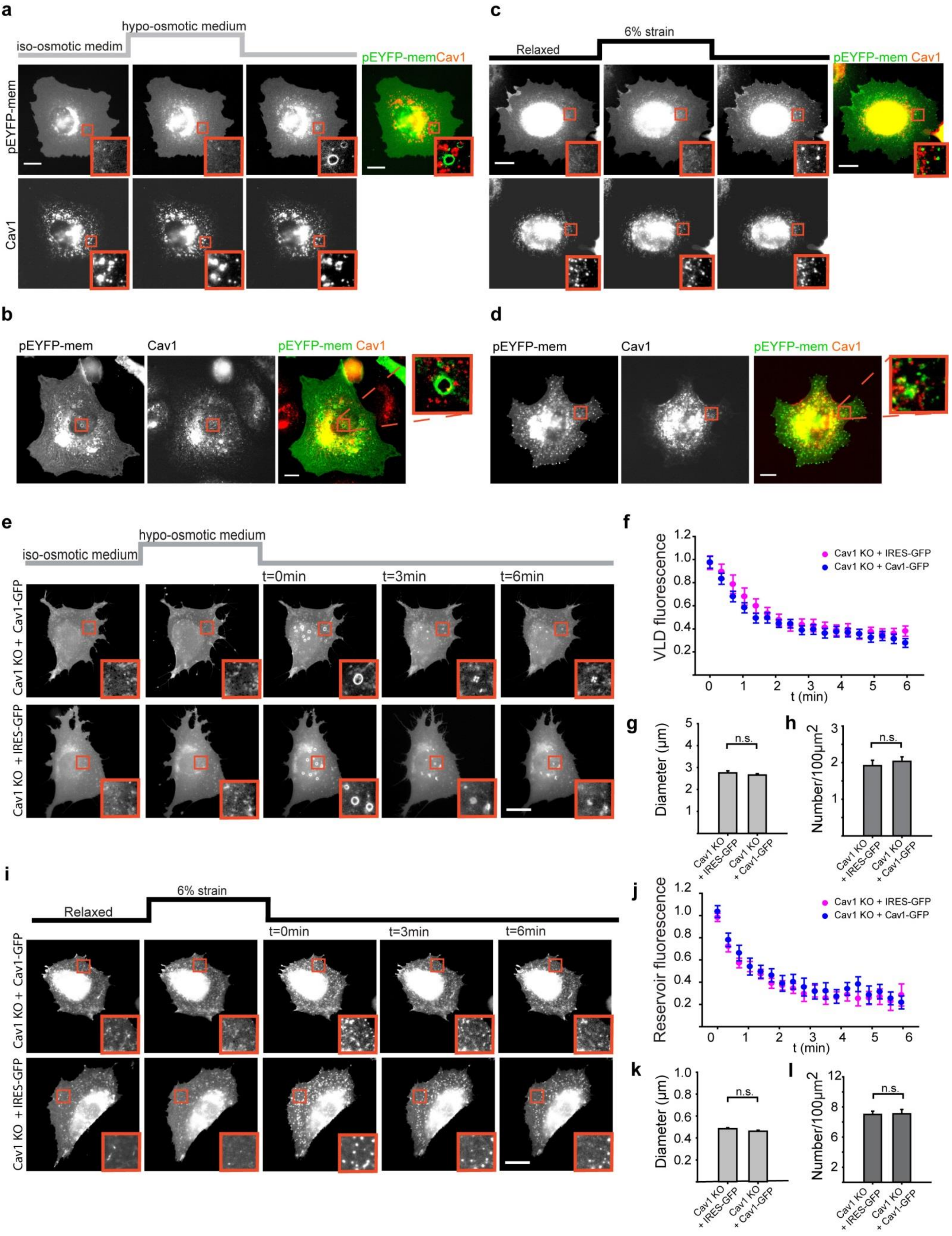

Supplementary Figure 6. Reservoirs and VLDs are unrelated to caveolae. a, Time sequence of pEYFP-mem and Cav1-mcherry transfected cells before, during, and after application of 6% stretch for three minutes. Image to the right shows the lack of co-localization between pEYFP-mem (green) and Cav1-mcherry (red). **b,** pEYFP-mem transfected cell (green in merged image) fixed right after restoring iso-osmotic media and stained for caveolin 1 (red in merged image). No co-localization was observed between caveolin and VLDs. **c**, Time sequence of pEYFP-mem and

Cav1-mcherry transfected cells before, during, and after application of 50% hypo-osmotic media for three minutes. Image to the right shows the lack of co-localization between peYFP-mem (green) and Cav1-mcherry (red). **d**, pEYFP-mem transfected cell (green in merged image) fixed right after releasing 6% stretch and stained for caveolin 1 (red in merged image). No co-localization was observed between caveolin and reservoirs. **e**, Time sequence of pEYFP-mem transfected cells before, during, and after application of 50% hypo-osmotic media during three minutes. Zoomed insets show the formation and evolution of VLDs. Caveolin 1 knock-out cells were reconstituted either with caveolin 1-GFP or an empty control vector (IRES-GFP). **f**, Quantification of VLD fluorescence after re-application of iso-osmotic media (1: initial fluorescence, 0: background). n=50/25 VLDs from 5/3 cells. **g-h**, Corresponding quantification of mean VLD diameter (**g**) (n=100/60 VLDs from 5/3 cells) and density (**h**) (n=30/20 regions from 5/3 cells).No significant differences were observed. **i**, Time sequence of peYFP-mem transfected cells before, during, and after application of constant 6% stretch during three minutes. Zoomed insets show the formation and evolution of membrane reservoirs. Caveolin 1 knock-out cells were reconstituted either with caveolin 1-GFP or an empty control vector (IRES-GFP). **j**, Quantification of reservoir fluorescence after stretch release (1: initial fluorescence, 0: background) (n=30/60 reservoirs from 3/4 cells) . **k**, Corresponding quantification of mean reservoir diameter (n=250/100 reservoirs from 3/3 cells). No significant differences were observed. **l** , Corresponding quantification of mean reservoir density (n=40/20 regions from 3/3 cells). No significant differences were observed. Scale bars are 20μm.

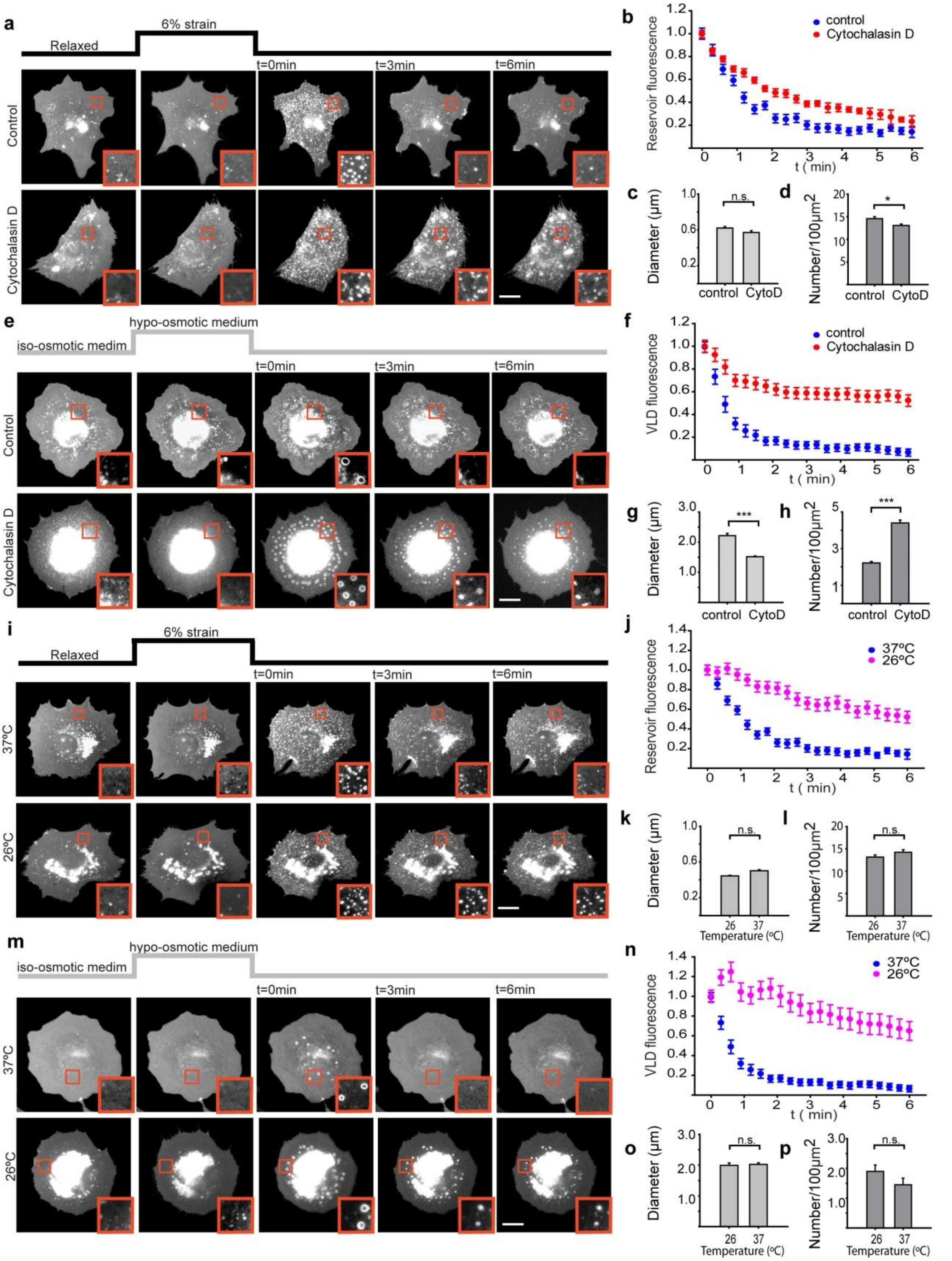

Supplementary Figure 7. Actin and temperature dependence of reservoirs and VLDs. a, Time sequence of peYFP-mem transfected cells before, during, and after application of constant 6% stretch during three minutes for control cells and cells treated with 0.5 μM cytochalasin D. Zoomed insets show the formation and evolution of membrane reservoirs. **b**, Quantification of reservoir fluorescence after stretch release (1: initial fluorescence, 0: background)

(n=100/100 reservoirs from 10/4 cells). **c**, Corresponding quantification of mean reservoir diameter (n=250/100 reservoirs from 8/4 cells). No significant differences were observed. **d**, Corresponding quantification of mean reservoir density (n=30/30 regions from 5 cells). **e**, Time sequence of peYFP-mem transfected cells before, during, and after application of 50% hypo-osmotic media during three minutes for control cells and cells treated with 0.5 µM cytochalasin D. Zoomed insets show the formation and evolution of membrane VLDs. **f**, Quantification of VLD fluorescence after re-application of iso-osmotic media (1: initial fluorescence, 0: background). n=100/100 VLDs from 10/5 cells. **g**, Corresponding quantification of mean VLD diameter (n=100/60 VLDs from 10/3 cells). **h**, Corresponding quantification of mean VLD density (n=50/30 regions from 8/3 cells). **i**, Time sequence of peYFP-mem transfected cells before, during, and after application of constant 6% stretch during three minutes for control cells at 37º and cells at 26º. Zoomed insets show the formation and evolution of membrane reservoirs. **j**, Quantification of reservoir fluorescence after stretch release (1: initial fluorescence, 0: background) n=100/60 reservoirs from 10/3 cells. **k,** Corresponding quantification of mean reservoir diameter (n=250/140 reservoirs from 8/3 cells). No significant differences were observed. **l**, Corresponding quantification of mean reservoir density (n=30/30 zones from 5/3cells)**.** No significant differences were observed. **m**, Time sequence of peYFP-mem transfected cells before, during, and after application of 50% hypo-osmotic media during three minutes for control cells at 37º and cells at 26º. Zoomed insets show the formation and evolution of membrane VLDs. **n**, Quantification of VLD fluorescence after re-application of iso-osmotic media (1: initial fluorescence, 0: background). n=100/24 VLDs from 10/3 cells. **o**, Corresponding quantification of mean VLD diameter (n=100/45 VLDs from 10/3 cells). No significant differences were observed**. p,** Corresponding quantification of mean VLD density (n=50/25 zones from 8/3 cells). No significant differences were observed. Scale bars indicate 20 µm. N.s., non-significant, *, p<0.05, ***, p<0.001. Scale bars are 20 µm.

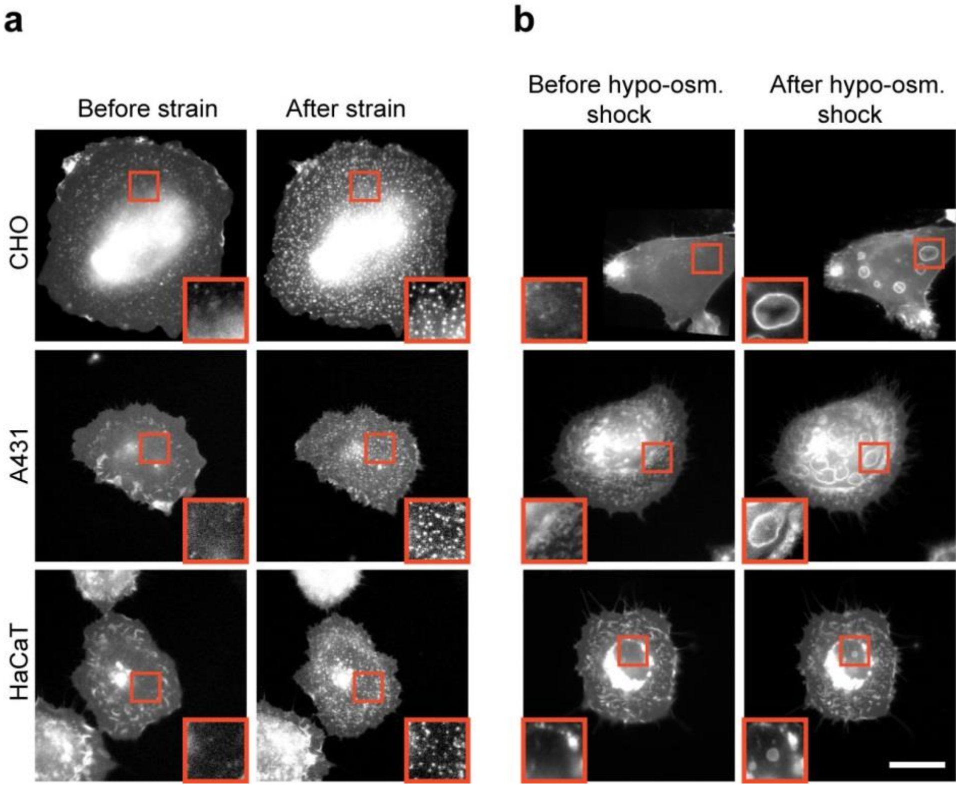

Supplementary Figure 8. Membrane reservoirs and VLDs are observed across cell types and species. Images of Chinese Hamster Ovary (CHO) cells, A431 human squamous carcinoma cells, and human keratinocytes (HaCaT) transfected with pEYFP-mem before and after being submitted to biaxial stretch (a) or hypo-osmotic shock (b). All cell types consistently showed the formation of reservoirs or VLDs, respectively. Zoomed insets (10x10 µm) show a magnification of both membrane structures. Scale bar indicates 20 µm.

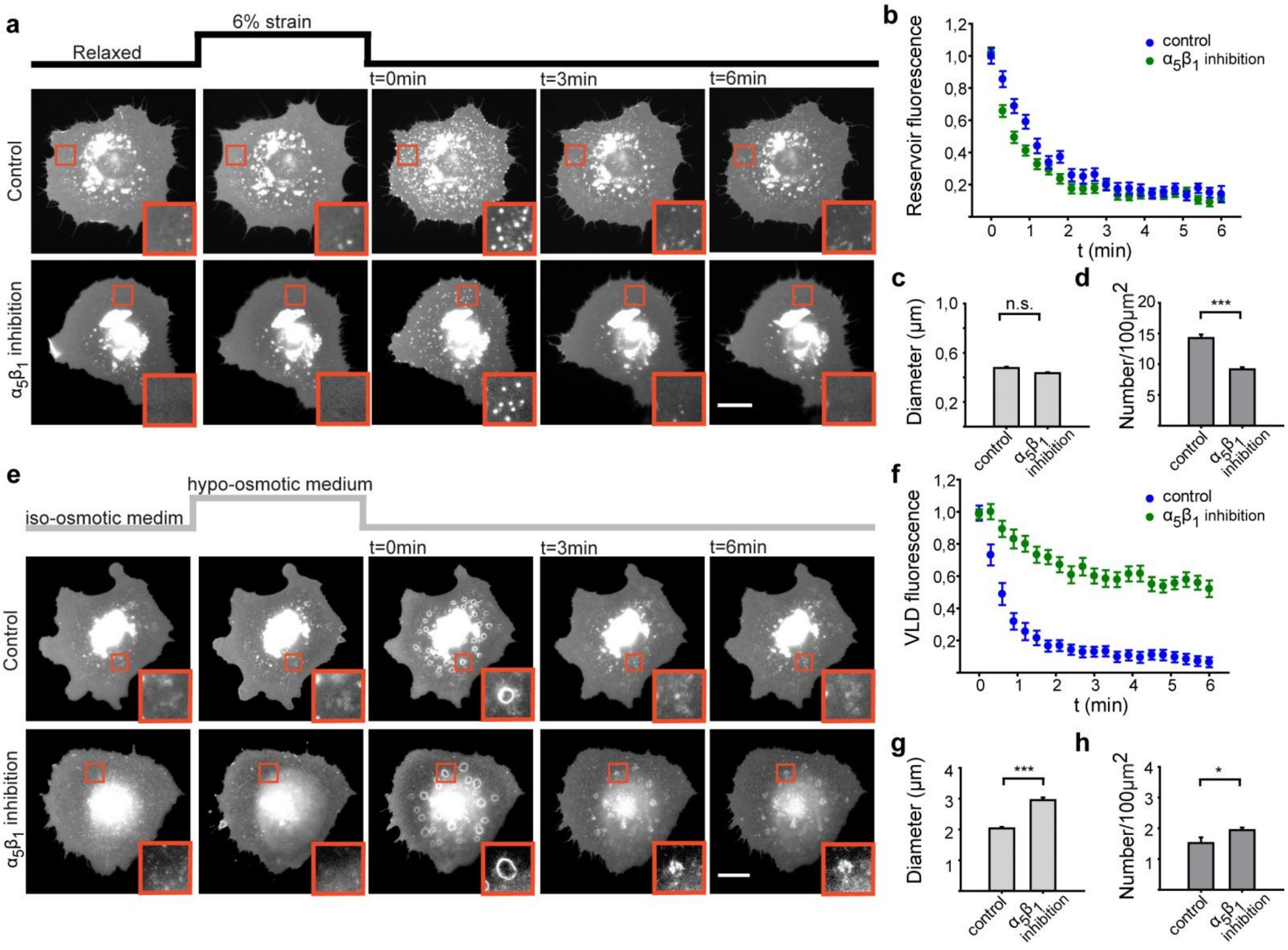

Supplementary Figure 9. Adhesion dependence of reservoirs and VLDs. **a**, Time sequence of pEYFP-mem transfected cells before, during, and after application of constant 6% stretch during three minutes for control cells and cells treated with 10 μg mL^{-1}α5β1 antibody. **b**, Quantification of reservoir fluorescence after stretch release (1: initial fluorescence, 0: background) (n=100/150 reservoirs from 10/6 cells). **c**, Corresponding quantification of mean reservoir diameter (n=250/200 reservoirs from 8/6 cells). **d**, Corresponding quantification of mean reservoir density (n=30/60 regions from 5/5 cells). **e**, Time sequence of pEYFP-mem transfected cells before, during, and after application of 50% hypo-osmotic media during three minutes for control cells and cells treated with 10 μg mL^{-1}α5β1 antibody. **f**, Quantification of VLD fluorescence after re-application of iso-osmotic media (1: initial fluorescence, 0: background). n=100/40 VLDs from 10/5 cells. **g**, Corresponding quantification of mean VLD diameter (n=100/150 VLDs from 10/7 cells). **h**, Corresponding quantification of mean VLD density (n=50/70 regions from 8/7 cells). Scale bars indicate 20 μm. N.s., non-significant, *, p<0.05, ***, p<0.001. Scale bars are 20 μm. In all cases, Zoomed insets (10x10 μm) show the formation and evolution of membrane structures.

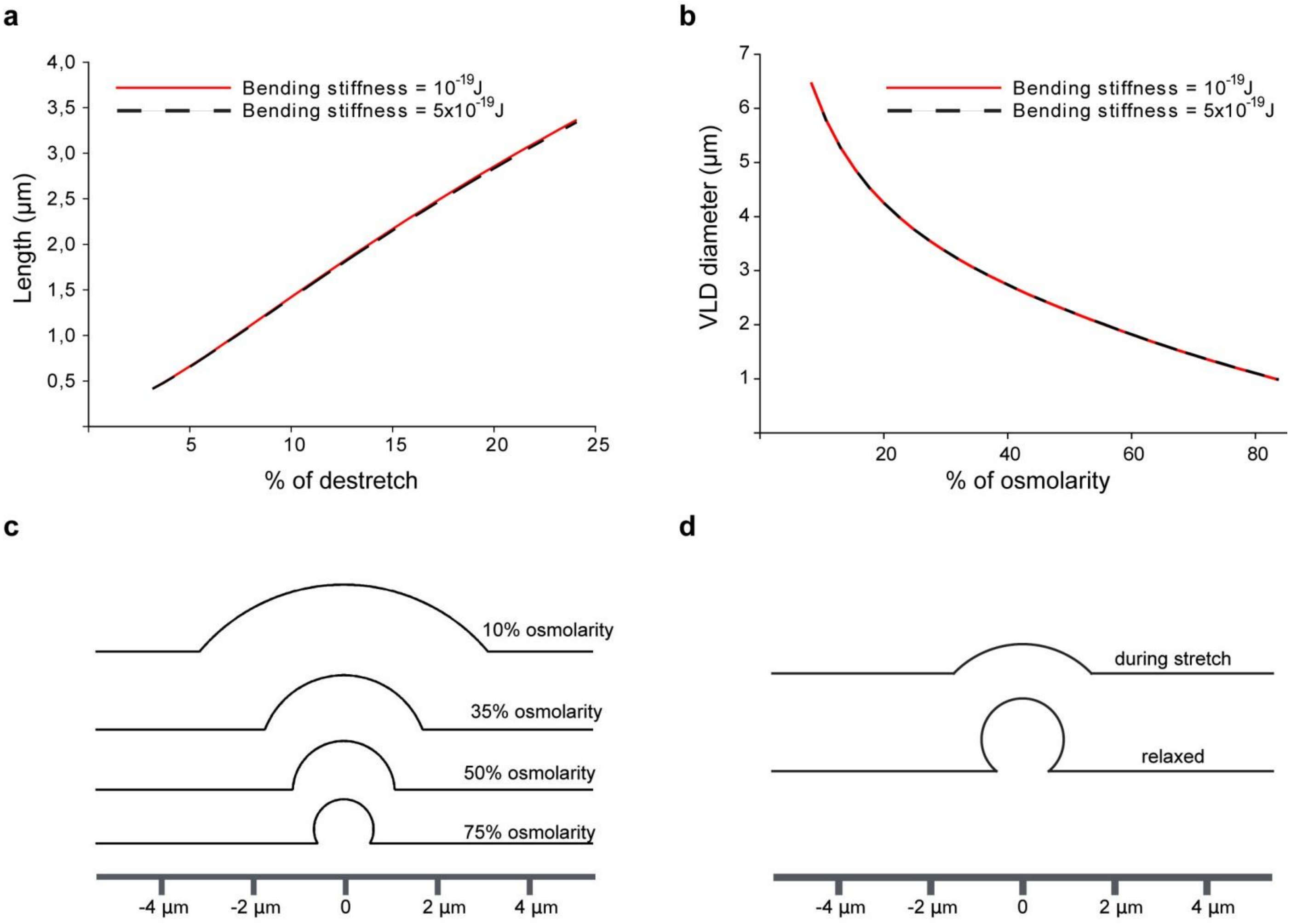

Supplementary Figure 10. Model predictions. **a**, Model prediction for reservoir length upon stretch release as a function of applied stretch. **b**, model prediction for VLD diameters formed after restoring iso-osmotic medium from osmotic shocks of different magnitude. In a and b, red line corresponds to the bending modulus used throughout the work, dashed black line shows the prediction for a 5-fold increase in bending modulus. **c**, VLD shape after restoring osmolarity from hypo-osmotic shocks of different magnitude and **d,** VLD shape after restoring osmolarity from a 50% hypo-osmotic shock in a pre-stretched membrane (top) and then de-stretching the membrane by 6% (top).

Supplementary Notes

Supplementary Note 1 – Dynamics of reservoir and VLD formation.

Throughout the figures, images of stretch-induced reservoirs and osmolarity-induced VLDs generally show fully formed structures at the first frame after their appearance. In the case of reservoirs, this is because their formation was faster than the few seconds required to re-position and re-focus cells after being moved by the stretch device. In the case of VLDs, a few seconds to re-focus after changing the medium were also required. This is because we used an upright microscope with a water-immersion objective that imaged through the medium, which lost focus after medium changes. Whereas the time lag required after stretch operations was unavoidable, we could eliminate the lag required after osmolarity changes by imaging with an inverted microscope. In this case, we observed a VLD formation time of approximately 20 s (Supplementary Figure 3a). Interestingly, the formation period of both reservoirs and VLDs became observable even with the upright microscope after either increasing the magnitude of stretch/hypo-osmotic shock or after decreasing cell activity by reducing temperature (Supplementary Figure 3b-c). This shows that the dynamics of VLD/reservoir formation are governed both by the magnitude of the applied stimulus and the active ability of the cell to recover its original state.

Supplementary Note 2 – Driving forces behind VLD formation and maintenance.

It is important to distinguish that VLDs form due to the need to store water volume at the cell-substrate interface, not due to changes in the volume of cells themselves. In earlier work, VLDs had been hypothesized to initiate as membrane invaginations driven by cellular shrinking, then enlarge due to hydrostatic pressure transients, and finally become stabilized by a spectrin cytoskeletal lining [1, 2, 3]. However, we believe that hydrostatic pressure of confined water at the cell-substrate interface accounts for all stages of the VLD cycle for different reasons. First, exposure to 50% osmotic shocks for 3 minutes only generates small membrane compressions of approximately 2%, which do not form any visible membrane invaginations (Supplementary Figure 2). Second, elimination of water confinement by using polyacrylamide gels abrogates VLD formation altogether. Finally, decreasing osmolarity after VLD formation collapses VLDs (Fig. 7e), showing that water flows are essential to their maintenance. Similarly, VLDs in cells with ATP depletion (Fig. 5e-f), cytochalasin D treatment (Supplementary Figure 7e-f), or reduced temperature (Supplementary Figure 7m-n) gradually collapse, but do not disappear entirely. This suggests that progressive flow from VLDs to the exterior bath reduces pressure and collapses the structure, while active resorption of membrane accumulation is impaired by the different treatments. Thus, hydrostatic pressure driven by confined water generates and maintains VLDs. A detailed analysis of the process would nevertheless require the consideration of the specific and non-specific adhesion between cells and their substrate, and the complex dynamics of confined flows through this interface.

Another important aspect to clarify is that the formation of VLDs, while driven by water volume storage, also requires the recruitment of membrane area. This is exemplified by the collapsed VLDs observed after either decreasing osmolarity (Fig. 7e), depleting ATP (Fig. 5e-f), treating with cytochalasin D treatment (Supplementary Figure 7e-f), or reducing temperature (Supplementary Figure 7m-n). In fact, collapsed VLDs are membrane accumulations akin to reservoirs, which like reservoirs can be eliminated through application of stretch (Fig. 7e).

Supplementary Note 3 – Model assumptions, parameters, and predictions.

We compare the experimental observations with the predictions of the model described in detail in[4]. In this model, a circular membrane patch with one protrusion at its center is considered, and the optimal shape is obtained by minimizing the energy of the system, consisting of elastic (stretching and bending) and adhesive contributions. Depending on the excess membrane area, given by the applied strain, and the interstitial volume, or alternatively the osmotic gradient across the membrane, various optimal shapes emerge, ranging from shallow caps when very little membrane area is available to very thin tubules when very little interstitial volume is available. The nominal separation between membrane structures is modeled through the size of this circular patch, and can be obtained from experiments. For instance, consistent with a density of between 1 and 2 VLDs per 100 μm^2, we choose a domain size of 5 µm. The higher density of reservoirs is modeled with a domain size of 1.25 µm.

For the adhesion energy, we use a cell-measured value of 4 mJ/m^2, reflecting the adhesion strength of a cell membrane to an RGD-coated substrate[5]. We use the same parameters as in[4] for the lipid bilayer stretching (K_s = 0.12 J/m^2) and bending moduli (k = 10^{-19} J). We note that unlike the model bilayer membranes in[4], cell membranes are lined with an actin cortex. This cortex has a stretching modulus of the order of 10^{-4} J/m^2, as obtained from a Young's modulus and thickness of the order of 10^3 Pa and 100 nm, respectively[6, 7]. This is three orders of magnitude below the value of the membrane itself, indicating that the contribution of the cortex to the overall stretching modulus is negligible. In contrast, the cortex would be expected to significantly increase membrane bending modulus. Indeed, it has been shown that cells with impaired membrane-actin links have a membrane bending stiffness comparable to that of lipid bilayers, whereas this bending stiffness increases 5-fold in cells with a normal membrane-cortex connection[8]. However, a 5-fold increase in bending stiffness did not significantly alter model predictions of the experimental observables, such as tube length or VLD diameter (Supplementary Figure 10a-b). Given the very high membrane stretching modulus and the strong osmolarity differences across the bilayer, the main factors determining the shape of tubes or VLDs were simply the excess area (compressive strain) and the volume enclosed in the interstitial space. Consistently with a negligible effect of bending stiffness, depolymerizing the actin cytoskeleton with cytochalasin did not impair reservoir or VLD formation (Supplementary Figure 7e and[1]), and barely affected reservoir diameter (Supplementary Figure 7c). The more marked effect of cytochalasin on VLDs (reduction in diameter of 30%, and a 2-fold increase in density, Supplementary Figure 7g) is probably due not to the cortex but to stress fibers and focal adhesions, through which VLDs have to make their way (Supplementary Figure 4). Cytochalasin treatment would reduce cytoskeletal resistance from focal adhesions and stress fibers, leading to the generation of many small VLDs throughout the cell. In contrast, increased cytoskeletal resistance in control cells would allow VLD formation only in specific sites with low resistance, which would then have to accommodate a larger water volume.

The only parameter that we adjust to fit our experimental results is the equilibrium separation of the adhesion potential, which in[4] was set to 3 nm. Here, best results are obtained with a value of 30 nm, reflecting the additional separation likely provided by integrins (extending approximately 20 nm from the membrane[9]) and the fibronectin coat. With these parameters, the model closely replicates the observations. For instance, the model predicts tubule length to increase linearly with applied destretch, see Fig. 6b. The VLD diameter predicted by the model for a 2% excess area and various degrees of osmolarity reduction from baseline osmolarity (M_0 = 300 mOsm) agrees well with the experimental quantification in Fig. 6c (see Supplementary Figure 10a). Mimicking Fig. 7g,j , increasing the membrane area by de-stretch after VLD formation in pre-stretched cells results in bud-like protrusions with a small neck and a slightly smaller apparent diameter, which store the same volume as the hemispherical VLDs but significantly more membrane area, see Supplementary Figure 10b. In agreement with Fig. 5p, we find that a three-fold increase in the density of VLDs (modelled through a corresponding decrease in the size of the membrane patch) results in a decrease of VLD diameter from 2.2 µm to 1.6 µm.

Supplementary references

1. Herring TL, Cohan CS, Welnhofer EA, Mills LR, Morris CE. F-actin at newly invaginated membrane in neurons: implications for surface area regulation. *J Membr Biol* 1999, **171**(2)**:** 151-169.

2. Herring TL, Juranka P, McNally J, Lesiuk H, Morris CE. The spectrin skeleton of newly-invaginated plasma membrane. *J Muscle Res Cell Motil* 2000, **21**(1)**:** 67-77.

3. Morris CE, Homann U. Cell surface area regulation and membrane tension. *J Membr Biol* 2001, **179**(2)**:** 79-102.

4. Staykova M, Arroyo M, Rahimi M, Stone HA. Confined Bilayers Passively Regulate Shape and Stress. *Physical Review Letters* 2013, **110**(2)**:** 028101.

5. Rico F, Roca-Cusachs P, Sunyer R, Farre R, Navajas D. Cell dynamic adhesion and elastic properties probed with cylindrical atomic force microscopy cantilever tips. *Journal of Molecular Recognition* 2007, **20**(6)**:** 459-466.

6. Clark AG, Dierkes K, Paluch EK. Monitoring actin cortex thickness in live cells. *Biophys J* 2013, **105**(3)**:** 570-580.

7. Salbreux G, Charras G, Paluch E. Actin cortex mechanics and cellular morphogenesis. *Trends Cell Biol* 2012, **22**(10)**:** 536-545.

8. Simson R, Wallraff E, Faix J, Niewohner J, Gerisch G, Sackmann E. Membrane bending modulus and adhesion energy of wild-type and mutant cells of Dictyostelium lacking talin or cortexillins. *Biophys J* 1998, **74**(1)**:** 514-522.

9. Eng ET, Smagghe BJ, Walz T, Springer TA. Intact αIIbβ3 Integrin Is Extended after Activation as Measured by Solution X-ray Scattering and Electron Microscopy. *J Biol Chem* 2011, **286**(40)**:** 35218-35226.
